# A syncing feeling: reductions in physiological arousal in response to observed social synchrony

**DOI:** 10.1093/scan/nsaa116

**Published:** 2020-08-25

**Authors:** Haley E Kragness, Laura K Cirelli

**Affiliations:** Department of Psychology, University of Toronto Scarborough, Toronto, Canada; Department of Psychology, University of Toronto Mississauga, Mississauga, Canada; Department of Psychology, University of Toronto Scarborough, Toronto, Canada

**Keywords:** synchrony, skin conductance, physiology, movement, interpersonal

## Abstract

Synchronized movements are often key elements in activities where social bonding and emotional connection are a shared goal, such as religious gatherings, sporting events, parties and weddings. Previous studies have shown that synchronous movements enhance prosocial attitudes and affiliative behaviors. Similarly, observers attribute more social closeness to people moving synchronously together than people moving asynchronously. The mechanisms by which synchrony modulates these attributions are not well understood. In the present study, we ask whether viewing synchronous activities influences physiological arousal as measured by skin conductance and whether group size impacts this effect. Undergraduates viewed a series of short videos depicting people moving either (1) in or out of synchrony with each other and (2) in a large or small group. Participants’ skin conductance was measured. Change in skin conductance levels and response counts were attenuated while watching synchronous movement, but only in the large-group condition. Post-hoc analyses suggest that viewer enjoyment/interest in the large-group synchronous videos mediated this association for phasic skin conductance responses, but no evidence of mediation was found for tonic skin conductance levels. Results extend previous research on affiliative effects of first-person interpersonal synchrony and demonstrate that watching others moving synchronously has an attenuating effect on observers’ physiological state.

## Introduction

From birth and throughout the lifespan, humans are particularly drawn to social interactions. We interact directly with others on a daily basis, and as third parties, observe numerous social interactions. These interactions convey complex information about social relationships to actors and observers through facial expressions, language and prosody, gestures and interpersonal movements.

The way that people move while interacting both reflects and influences social evaluations and goals. For instance, adults subconsciously mimic others’ movements, particularly when interacting with someone they like or need to coordinate with to achieve a shared goal (Chartrand and Lakin, 2013). Co-actors’ body sway becomes coupled during activities that require coordination, such as in conversation and group music performance (Shockley *et al.*, 2009; Chang *et al.*, 2019). The social implications of movement dynamics are present early in development—behavioral mimicry can be observed in children as young as 3 years old (van Schaik and Hunnius, 2016) and infants become upset when maternal responses are misaligned and therefore noncontingent with their movements (Bigelow *et al.*, 1996; Moore *et al.*, 2009).

Interpersonal synchrony is a specific form of movement coordination that has captured recent attention, especially that of social, music cognition and developmental psychologists. Here, we define interpersonal synchrony as time-locked movements between two or more individuals. This can be achieved by, for instance, dancing, marching, rowing or singing in synchrony with others. In naturalistic settings, interpersonal synchrony often involves a series of movements that are rhythmic and predictable. In contrast to mimicry, the timing of movements must be closely aligned, and the nature of the movements themselves is less important than timing.

Experiencing interpersonal synchrony firsthand has been shown to facilitate social bonding and prosociality. Adults rate one another as more likeable, and help, trust and cooperate with one another after moving synchronously compared with asynchronously (Hove and Risen, 2009; Wiltermuth and Heath, 2009; Kokal *et al.*, 2011). These effects emerge early in life. For example, 12-month-old infants are more likely to seek proximity with (Tunçgenç *et al.*, 2015) and 14-month-old infants are more helpful toward previously synchronous compared to asynchronous partners (Cirelli *et al.*, 2014a; Cirelli, 2018). Young children are more helpful and cooperative with synchronously moving peers over asynchronously moving peers (Tunçgenç and Cohen, 2016; Rabinowitch and Meltzoff, 2017) and rate these individuals as more similar to themselves (Rabinowitch *et al.*, 2015). In general, experiencing synchrony appears to specifically promote prosocial behavior toward those involved in the synchronous activities (Kokal *et al.*, 2011; Cirelli *et al.*, 2014b; Tarr *et al.*, 2015; Cross *et al.*, 2019a), supporting the group formation framework (Cross *et al.*, 2019b).

Various mechanistic theories have been proposed, spanning social-cognitive, perceptual, neurohormonal, and evolutionary perspectives. Social-cognitive theories have outlined the ways in which synchrony may blur the lines between the sense of self and other (Valdesolo and DeSteno, 2011; Cross *et al.*, 2019b). Perceptual theories suggest that our attention is directed toward synchronously moving others, encouraging increased person perception (Macrae *et al.*, 2008; Kokal *et al.*, 2011). From a neurohormonal perspective, experiencing synchrony has been linked to increased endorphin release (Launay *et al.*, 2016) and may be enhanced by oxytocin (Gebauer *et al.*, 2016; Shamay-Tsoory *et al.*, 2019). Little work has explored the brain regions that are involved when experiencing synchrony, but preliminary reports suggest involvement of the caudate and the brain’s reward system (Kokal *et al.*, 2011), and that synchronous movement is associated with interbrain synchrony (Hu *et al.*, 2017). Evolutionary theories suggest that synchronous movement (especially when achieved by drumming, singing or dancing together) strengthens social bonding, therefore providing group-level benefits by fostering intergroup cooperation (Brown, 2000; Hagen and Bryant, 2003; Reddish *et al.*, 2013).

While most previous work has focused on first-hand experiences of synchronous movement, a separate but related line of work has examined third-party evaluations of synchronous movers. To our knowledge, however, no studies have investigated how an observer’s internal state is affected. Measurement of physiological arousal is one reflection of internal states. States of heightened arousal are associated with desire to act (e.g. fight-or-flight response), and states of lowered arousal are associated with relaxation (e.g. preparing for sleep).

By one view, observing synchrony may signal group formidability, which could increase observer arousal. Synchronous movements have been proposed to act as a ‘coalitional signaling’ system to other groups, establishing the synchronous group as strong, capable and coordinated (Hagen and Bryant, 2003). Indeed, hearing the sound of a group’s synchronous compared with asynchronous footsteps biases listeners to perceive the members of the synchronous groups as more formidable (Fessler and Holbrook, 2016). If third-party synchrony communicates group strength (‘coalitional signaling hypothesis’), observers should experience elevated arousal in response to such displays, and this effect should be more pronounced as group size grows.

A contrasting hypothesis (‘affiliative others hypothesis’) is that the observers’ internal state would be affected by the inferred relationship between the co-actors in the video. For example, by as early as 12 months of age, infants expect synchronous movers to interact prosocially and seek proximity with one another more so than asynchronous movers (Cirelli *et al.*, 2014b; Fawcett and Tunçgenç, 2017). Adults assume that characters waving their hands in synchrony are more similar and more likely to behave as a social unit than characters waving asynchronously (Lakens, 2010; Lakens and Stel, 2011). When observing or hearing others walking in synchrony *vs* out-of-step, synchronous movers are also rated to be higher in rapport (Miles *et al.*, 2009; Edelman and Harring, 2015), higher in cohesiveness, and more likely to have a common goal (Wai-man *et al.*, 2006). Given the body of research showing that observers attribute affiliation between synchronous others, thereby reducing the potential for social conflict, this framework would predict lower arousal when observing synchrony than asynchrony.

While a number of studies have examined observers’ judgments about synchronous *vs* asynchronous others, to our knowledge, no work has examined potential effects on the observer’s own internal state. Here, we presented adults with short silent videos of small or large groups of people moving either in- or out-of-synchrony with one another. While observers watched these videos, we continuously recorded their arousal levels, as indexed by skin conductance levels (SCL, tonic activity), the smooth and slow-varying skin conductance signal, and skin conductance responses (SCRs, phasic activity), which reflect rapid fluctuations in electrodermal activity. We expected that physiological arousal would be modulated by both synchrony and size of the observed group.

## Methods

### Participants

The final participant sample consisted of 46 young adults (*M* age = 18.91 years, s.d. = 1.55, 29 women, 1 left-handed). An additional 14 participants were excluded due to equipment failure (*N* = 4) or excessively noisy physiological recordings (*N* = 10). Twenty-seven of the 46 participants reported a history of music training. The average years of training among all participants was 3.07 years (median = 2, s.d. = 3.97, range = 0–13 years), and 18 reported a history of dance training (*M* years of training = 1.91, median = 0, s.d. = 3.75, range = 0–17 years). Participants received course credit for participating. The University of Toronto Research Ethics Board approved all data collection procedures, and informed consent was obtained from all participants.

### Apparatus

Testing took place in a double-walled sound-attenuating booth (Industrial Acoustics). BIOPAC MP35 in conjunction with Acq-Knowledge 5.0 software running on a Windows 10 computer recorded participant SC at a sampling rate of 100 Hz. Two pre-geled, self-adhesive, Ag–AgCl electrodes were connected via leads to the MP35 system. Electrodes were placed on the distal phalanx of the index and middle fingers of the participants’ nondominant hand. Video of the stimulus presentation screen was recorded through AcqKnowledge with Sony Exmor R camcorders in order to align physiology with participant experience.

### Stimuli

**Fig. 1.**
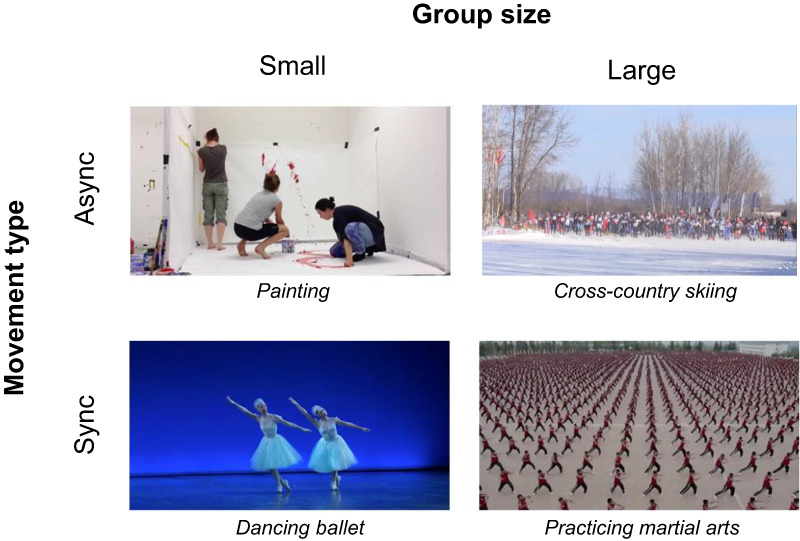
Depicts an representative screenshot from one video in each condition. In the full experiment, there were 12 videos per combination of conditions, for a total of 48 trials.

Stimuli consisted of silent videos downloaded from YouTube (stimuli are available on the Open Science Framework, https://osf.io/pr3td/). The videos were of either small (2–3) or large (≥6) groups of people engaged in either synchronous or asynchronous movement. For the purposes of this study, ‘synchronous’ movement was considered to be identical (synchronized swimmers performing the same gesture at the same time) or perfectly mirrored versions of the same movement (people rowing on the left *vs* right side of a canoe in a synchronized manner). Videos that featured people moving in time with coordinated movements that were not identical (for example, a musical quartet playing together in time using different instruments and different gestures), were not included. Two videos included camera angle changes (‘S_S_TandemCycling’ and ‘S_L_KungFuAcademy’), but all other videos were single-shot (one camera). We avoided including videos that were likely to elicit strong preexisting emotional connotations due to familiarity (e.g. famous events, well-known people or sports teams, etc.). Twelve videos were selected for each combination of conditions, for a total of 48 videos (see Figure 1 for examples).

### Procedure

Participants were told they would be watching a series of short videos while their physiological arousal was recorded. They were informed that after each video, they would be asked to rate (1) how ‘in sync’ the people were when doing the main activity (1 = not at all to 5 = perfectly), (2) approximately how many people were doing the main activity (1 = 1–3 people to 8 = >1000 people) and (3) how much training and/or expertise would be required to perform the task featured in the video (1 = none, 0 years to 5 = extreme, >10 years). This was done both as a manipulation check and to encourage participants to attend to the videos (see Supplemental Material for a discussion of participants’ ratings). The experimenter told the participants to answer to the best of their ability, but further, that they should not spend time actively counting each person in the video. Instead, they should passively watch the video and answer the questions quickly using their impressions of what they saw. The video portion of the experiment took approximately 35 to 50 minutes, varying based on how quickly they made their responses.

The participants were additionally asked to fill out a demographics questionnaire, which asked about language and music experiences, as well as the Interpersonal Reactivity Index (IRI) (Davis, 1980) to assess facets of empathy. Participants filled out the demographics questionnaire after the electrode application, but before the video-watching phase. This allowed the signal to stabilize and gave the experimenter the opportunity to make adjustments to the electrodes prior to the video phase, if needed. Participants filled out the IRI after the video-watching portion.

Finally, they were asked to report their belief about the purpose of the study. Only 3 of the 46 participants’ responses included linking synchrony to physiological arousal. The participants were debriefed and any remaining questions were answered by the experimenter.

### Data processing

SC data were exported into MATLAB 2016a and then further processed in the Ledalab (V3) toolbox. Data were down sampled to 10 Hz, and minor artifacts were visually identified and corrected. Trials with extreme artifacts were identified and excluded from further analyses (<0.01% of trials).

Continuous Decomposition Analysis was used in Ledalab to extract tonic and phasic SC (Benedek and Kaernbach, 2010). The tonic component of the electrodermal signal (skin conductance level, SCL) is thought to reflect general arousal (e.g. Dawson et al., 2016; Braithwaithe *et al.*, 2013). Baseline SCL is known to drift over the course of an experiment and varies widely across individuals and measurement tools. For these reasons, here, we examined change in SCL over a trial, represented by the slope of the linear line of best fit from trial onset to trial end (see also Cirelli *et al.*, 2020 for this data processing procedure). The phasic component of the signal (skin conductance response, SCR) reflects rapid fluctuations above baseline in the electrodermal signal elicited in response to events of psychological or physiological significance, including novelty, aversiveness, social significance, emotional significance, and more (e.g. Dawson *et al.*, 2016; Braithwaithe *et al.*, 2013). Ledalab SC response reports were generated, identifying the number of SC responses exceeding a threshold of 0.1 microsiemens occuring between 1 and 18s after stimulus onset. One video (‘A_S_PartnerBouldering’) elicited both the highest average tonic slope and greatest average number of SCRs, and exceeded three s.d. from the group mean for both measures. Therefore, trials containing this video were excluded from all analyses. One participant’s data were excluded from the phasic analysis because visual inspection suggested their data were contaminated by high-frequency noise in the signal, which did not affect their tonic data.

### Analyses

Analyses were performed in R (v3.6.3, R Core Team, 2020). Comparisons were carried out using mixed-effects models with the lme4 package (Bates *et al.*, 2015), and significance was assessed with the lmerTest package (Kuznetsova *et al.*, 2017). Contrasts were selected for the fixed effect group size (large group was coded as 1 and small group as −1) and for movement type (synchronous was coded as 1 and asynchronous as −1), and random intercepts were included for participants and stimuli.

## Results

### Change in SCL (tonic slope)

Results indicated that neither group size nor movement type significantly predicted the change in SCL (*P = *0.449 and 0.609, respectively). The interaction between group size and movement type was significant (β* *= −0.0014, *SE* = 0.0005, *t* = −2.572, *P *= 0.014). To investigate this interaction, separate mixed-effect models were run for the effect of movement type within each group size condition. Movement type significantly predicted tonic slope in the large-group condition (β* *= −0.0016, *SE* = 0.0005, *t* = −3.030, *P *= 0.003), but not the small-group condition (β* *= 0.0011, *SE* = 0.0009, *t* = 1.165, *P *= 0.257).

### Number of nonspecific SCRs (phasic component)

**Fig. 2.**
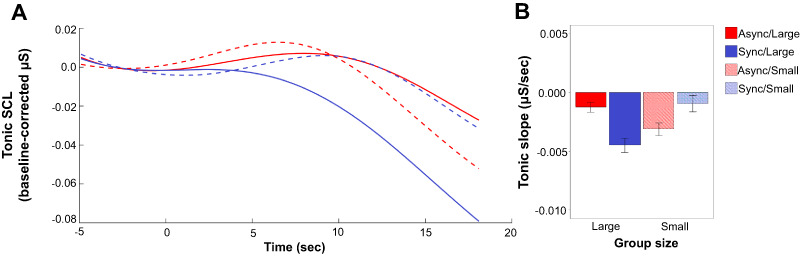
Tonic skin conductance levels while watching videos. A) This shows the skin conductance levels across the course of the videos (18 seconds). Here, skin conductance levels are baseline-corrected to the mean of the 5-second time window directly preceding trial onset. B) This depicts the mean tonic slope. Error bars indicate within-subject SEM (Cousineau, 2005).

**Fig. 3.**
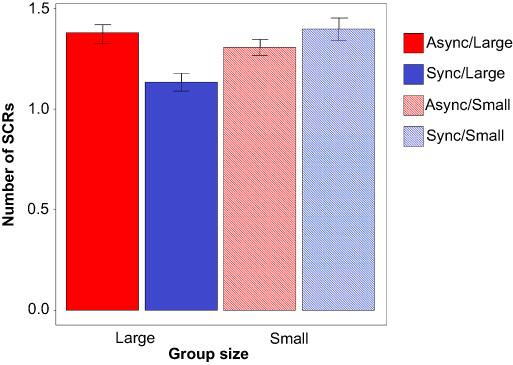
Phasic skin conductance responses while watching videos. Error bars represent within-subject SEM (Cousineau, 2005).

A generalized linear mixed effects model (LMEM) with a Poisson distribution was fit to examine the fixed effects group size and movement on the number of SCRs (see Figure 2), including participant and video as random effects (intercept). Neither group size nor movement type were significant predictors (*P *= 0.277 and 0.304, respectively), but there was a marginally significant interaction between group size and movement type (β* *= −0.064, *SE* = 0.033, *z* = −1.949, *P *= 0.051). Separate generalized mixed effects models for large and small group size were conducted to investigate the interaction. Results mirrored that of the tonic slope analysis: movement type was a significant predictor of the SCR count in the large-group condition β* *= −0.098, *SE* = 0.037, *z* = −2.606, *P *= 0.009, but not in the small-group condition, β* *= 0.030, *SE* = 0.054, *z* = 0.546, *P *= 0.585 (Figure 3 and Table 1).

**Table 1.** Mixed effects analyses for the tonic and phasic components

Effect	Estimate	*SE*	*t/Z*	*P*
**Change in SCL (tonic component)**				
**(Intercept)**	−0.0024	0.0007	−3.469	0.001
Group size	−0.0004	0.0005	−0.764	0.449
Movement type	−0.0003	0.0005	−0.515	0.609
Group size × movement type	−0.0014	0.0005	−2.572	0.014
**Number of nonspecific SCRs (phasic component)**				
**(Intercept)**	0.158	0.070	2.256	0.024
Group size	−0.036	0.033	−1.087	0.277
Movement type	−0.034	0.033	−1.028	0.304
Group size × movement type	−0.064	0.033	−1.949	0.051

Contrast coding: group size (large = 1, small = −1) and movement type (synchronous = 1, asynchronous = −1).

### Secondary analyses

The previous analyses demonstrated in two measures of physiological arousal that arousal was lowest when watching synchronous groups, but only in the context of large group sizes. However, the mechanism by which synchrony reduced physiological responses was not clear. In an exploratory analysis, we normed the data on several different dimensions that were hypothesized to mediate the association between viewing synchrony and decreased arousal. Based on the previous literature, we asked participants to rate each video on the following dimensions (1—not at all to 9—extremely):

1. How enjoyable was the video to watch? *(viewer enjoyment)*
2. How energetic was the activity in the video? *(energy of the video subjects)*
3. How interesting was the video to watch? *(viewer interest)*
4. How much do you think the people in the video like each other? *(perceived affiliation between video subjects)*
5. How similar do you feel to the people in the video? *(self-other similarity)*

The videos were normed by a sample of 36 undergraduate participants from the University of Toronto (mean age = 19.22 years, s.d.* *= 1.46, 28 women, 7 men, 1 prefer not to say). Participants did the experiment online at their leisure. The experiment was administered online through the Pavlovia platform (pavlovia.org). The experiment took approximately 30 minutes, and participants received course credit.

The ‘mediation’ package in R (Tingley *et al.*, 2019) was used to evaluate whether these five ratings significantly mediated the association between synchronous movement and each physiological arousal measurement in the large-group condition. Each possible mediator was tested separately, and unstandardized indirect effects were computed for 1000 nonparametric bootstrapped samples with a 95% confidence interval with the seed set to 2020. Results are summarized in Table 2. For tonic slope, none of the rated dimensions were significant mediators. For the phasic component, two mediators were significant—‘viewer enjoyment’ and ‘viewer interest’ completely mediated the association between movement type and SCR’s. ‘Enjoyment’ and ‘interest’ ratings for each video were highly correlated (Pearson’s *r* = 0.976, *P* < 0.0001, *n* = 24).

**Table 2.** Unstandardized indirect effects computed by bootstrapping 1000 samples

Effect	Unstandardized indirect effect (95% CI)	*P*
**Change in SCL (tonic component)**		
Viewer enjoyment	−0.0007 (−0.0020, 0.0005)	0.18
Video subject energy	−0.0004 (−0.0019, 0.0013)	0.59
Viewer interest	−0.0005 (−0.0017, 0.0008)	0.41
Video subject affiliation	−0.0006 (−0.0026, 0.0007)	0.45
Self-other similarity	−0.00007 (−0.0008, 0.0007)	0.88
**Number of nonspecific SCRs (phasic component)**		
Viewer enjoyment	−0.06 (−0.27, −0.013)	0.02
Video subject energy	−0.098 (−0.234, 0.036)	0.13
Viewer interest	−0.102 (−0.232, −0.006)	0.04
Video subject affiliation	−0.150 (−0.359, 0.029)	0.12
Self-other similarity	−0.021 (−0.151, 0.053)	0.57

## Discussion

Previous reports have shown that watching others move in synchrony *vs* asynchrony affects observers’ judgments about their social relationships. To our knowledge, no previous research has examined how watching synchronous movements affects the internal state of the observer. In the present study, we found that observing large groups moving in synchrony compared with asynchrony lowered physiological arousal levels, represented both by decreased tonic slopes and fewer phasic skin conductance responses. Group size played an important role across both measures, as the effect of synchrony was not observed for small group sizes.

The coalitional signaling hypothesis predicts that participants would view synchronously moving groups as stronger than asynchronously moving groups, resulting in a stress response and increased arousal. The observed reduction in arousal levels in response to synchrony did not support this hypothesis. One potential alternative hypothesis presented above, the ‘affiliative others’ hypothesis, is partially consistent with this pattern of data. Observing a cohesive interaction that is not likely to generate social conflict would conceivably facilitate a less vigilant physiological state. However, post hoc analyses suggested that the observed attenuation in physiological arousal could not be attributed to a mediating effect of perceived affiliation between the co-actors.

In the present work, the implementation of the coalitional signaling hypothesis assumes that the observer neither has nor simulates preexisting social relationships with the subjects of the video. However, it is possible that viewers felt socially connected to the actors in the video and that this modulated their arousal response. Work in social cognitive neuroscience has highlighted the role of neural ‘mirror’ systems during action observation (e.g. Rizzolatti and Fabbri-Destro, 2008), which suggest that action observation triggers action simulation through activation in motor areas of observers. It is possible that observers in the present experiment simulated their own participation in the observed action and experienced affiliative feelings toward the actors in the videos. Such affiliative feelings toward the actors could explain the observers’ more relaxed physiological state. If observers were presented with synchronous others from an apparent out-group, the formidability of those actors may then be perceived as threatening. However, given prior evidence for positive associations between the mirror neuron system, motor imitation and empathy (Baird *et al.*, 2011; Novembre *et al.*, 2019), we might expect that participants with higher empathy would be affected more strongly by watching synchronous *vs* asynchronous activities, which was not observed here (see Supplemental Material). ‘Self-similarity’ to the videos, as rated by undergraduates in a separate but similar sample, was also not a significant mediator for either synchrony effect, which does not provide evidence for this hypothesis. It should be noted, however, that the ratings used were ‘average’ self-similarity ratings (over a group), and that an individual-differences approach could better clarify the specific role of self-other similarity in this effect in the future.

Post hoc mediation analyses incorporating ‘self-other similarity’ and ‘perceived affiliation’ ratings were not fully consistent with either a ‘coalition signaling’ or ‘affiliative others’ hypothesis. Instead, results showed that ‘viewer enjoyment’ and ‘viewer interest’ mediated the synchrony effect on phasic SCRs, such that more enjoyable/interesting videos resulted in fewer SCRs. This is consistent with past research finding that SCRs are elicited in response to aversive events (Dawson *et al.*, 2016), and points to a potential mechanism for enjoyment, perhaps via aesthetic appreciation, in the physiological attenuation reported here. However, neither enjoyment nor interest mediated the synchrony effect on tonic SCL. Additionally, it is not clear why a mediating effect of enjoyment/interest on synchrony would be isolated to the ‘large-group’ condition, given that there was no evidence that the small-group synchronous videos were rated on average to be less enjoyable (*P* > 0.6).

A related idea is that perceptual ease of processing may have contributed to the observed effects. Observing synchrony is more perceptually fluent than observing asynchrony. It is possible that such fluency could lead to the physiological effects found here. While little work has measured physiological responses to fluency, there is preliminary evidence for lower skin conductance response amplitude in response to high-fluency compared with low-fluency images (Forster *et al.*, 2016). To our knowledge, whether perceptual factors like visual fluency contribute to the prosocial effects of synchrony has not been tested.

The effect of observed synchrony on physiology was only salient in large-group contexts for both dependent measures. Large groups also exaggerate the effects of experienced synchrony. For example, individuals experience elevated pain thresholds for a longer duration after synchronous rowing in large groups (12 people) compared with pairs (Lewis and Sullivan, 2018). Meta-analytic evidence supports the finding that group size modulates prosocial behaviors and positive affect for individuals moving in synchrony with others, with larger groups increasing both (Mogan *et al.*, 2017). Such findings support the theory that the social effects of synchrony may be especially effective in large group contexts where one-on-one bonding rituals like grooming are too time-consuming (Launay *et al.*, 2016). The present study underscores the importance of group size in synchrony from the observers’ perspective as well as the actors’.

Synchrony and elevated physiological arousal tend to co-occur in rituals like dancing and recent work has found that both synchrony and physiological arousal affect social measures (Jackson *et al.*, 2018; Tarr et al., 2015). While future work is required to elucidate the links between physiological arousal, prosociality and synchrony, the present study demonstrates the importance of considering the observer in our frameworks of social synchrony and supports recent proposals that group size is an important consideration in studies of group synchrony. Understanding observers’ internal responses to synchrony may be especially important for clarifying the social-emotional role of movement synchrony in various art forms, such as dance and music performance.

## Supplementary Material

nsaa116_Supp
