# Supplementary material for: A syncing feeling: reductions in physiological arousal in response to observed social synchrony
Source: Soc Cogn Affect Neurosci. 2020 Aug 25;16(1-2):177–84. doi: 10.1093/scan/nsaa116 (PMC8208370; doi:10.1093/scan/nsaa116)
Supplement: nsaa116_Supp [file nsaa116_supp.zip › scan-20-010-File007.docx]

**Supplemental Material**

**Group size correction**

During stimulus selection, efforts were made to match the approximate numbers of actors in the synchronous and asynchronous movement conditions in each size condition. After data collection, we deemed it useful to obtain a more accurate estimate of the number of people in each video to examine whether any outliers could be driving the observed effect based on Group Size. A trained research assistant coded the number of people they observed in three frames of each video – the first frame (millisecond 1), the middle frame (millisecond 9000), and the final frame (millisecond 18,000). The mean of these three numbers was considered to be the “actual” group size in the video. Counting was done using free software DotDotGoose v1.2.0 (Ersts, 2019). The average group size was larger in the Sync Large than Async Large condition (mean SL = 925.18 people, mean AL = 186.08 people). To ensure this was not driving the observed Group Size by Movement Type interactions, we re-ran the analyses of mixed effects models excluding the two largest group videos in the Sync Large condition (excluding “S_L_KungFuAcademy” and “S_L_WeAreChant” videos), which made the average group size in Sync-Large more comparable to Async-Large at 164.43 people. The same significant effects were observed in both the skin conductance level (tonic slope) and skin conductance response (phasic count) measures.

**Individual differences**

We predicted that individual differences in empathy might facilitate the magnitude of the difference in response to viewing synchrony – for instance, by modulating the extent to which individuals imagined themselves to be a part of the viewed actions. We hypothesized that participants’ scores on the *fantasy* (FS) and/or *perspective-taking* (PT) subscales of the IRI would correlate with their response to observing synchrony. We operationalized the synchrony response as the difference in tonic slope between asynchrony vs. synchrony in the large condition, and the difference between skin conductance responses to asynchrony vs. synchrony on average. No significant correlations were observed with any subscores of the IRI (all Pearson’s *r*’s between -.173 and .177, all *p*’s > .244; see Supplemental Table 1).

| Supplemental Table 1  *Correlations with IRI subscores and sync effect* | | | | |
| --- | --- | --- | --- | --- |
|  | Fantasy scale | Empathic concern | Perspective taking | Personal distress |
| Mean *(SD)* | 16.76 *(5.64)* | 21.22 *(4.77)* | 16.02 *(4.46)* | 13.89 *(4.33)* |
| Correlation with SCL sync effect (async minus sync, large group only) | -.100 | -.060 | .173 | .016 |
| Correlation with SCR sync effect (async minus sync) | -.092 | -.039 | -.034 | .0177 |

**Ratings**

*Synchrony ratings*. Participants’ synchrony ratings (scale of 1-5) were submitted to a Group Size by Movement Type ANOVA. There was a significant interaction, *F*(1,45) = 29.32, *p* < .001, *η*^2^_G_ = .044. As expected, paired *t*-tests confirmed that within each Group Size condition, the Sync *video* was rated as more synchronous than the Async video (*t*_L_(45) = 32.72, *p* < .001; *t*_S_(45) = 37.62, *p* < .001). There was no evidence that Large Sync vs. Small Sync groups were perceived to be more “in sync” (*t*(45) = -1.679, *p* = .100), but the Large Async videos were perceived to be significantly more “in sync” than Small Async videos (*t*(45) = 6.157, *p* < .001). This is likely attributable to the general tendency to perceive random motion as having structure, known as *motion pareidolia* (Davidenko et al., 2017; Ramachandran & Anstis, 1985).

*Size ratings.* We examined whether participants were more likely to over- or under-estimate group size based on Movement Type. Since we did not ask participants to guess a specific number, we converted the trained research assistants’ counts to the same scale (1-8) that participants used in the ratings task. Because video selection for Small Group size was limited to 2-3 people, while the selection for Large Group was ≥ 6 people, we did not include the Small Group ratings in this analysis. We subtracted the actual size (scaled) from the participants’ size rating . Mean difference scores were below 0 (Large/Sync size difference mean = -0.50, *SD* = 0.43; Large/Async size difference mean = -0.15, *SD* = 0.518), indicating that participants consistently underestimated group size. A paired *t-*test showed that participants underestimated the group size more for groups in the Sync video than the Async video, *t*(45) = -6.451, *p* < .001.

*Expertise ratings.* Participants’ expertise ratings (scale of 1-5) were submitted to a Group Size by Movement Type ANOVA. There was a significant interaction, *F*(1,45) = 6.273, *p* = .016, *η*^2^_G_ = .012. Small Group Size videos were rated as requiring more expertise than large Group Size videos (*F*(1,45) = 79.53, *p* < .001, *η*^2^_G_ = .101) and Sync Movement Type videos were rated as requiring more expertise than Async (*F*(1,47) = 223.645, *p* < .001, *η*^2^_G_ = .270). The differences in expertise rating for Sync and Async were more exaggerated in the Large compared to the Small condition (*t*(45) = 2.505, *p* = .016).

**References**

Top of Form

Ersts,P.J.[Internet] DotDotGoose (version 1.5.1). American Museum of Natural History, Center for Biodiversity and Conservation. Available from https://biodiversityinformatics.amnh.org/open_source/dotdotgoose. Accessed on 2020-7-3.

Bottom of Form

Davidenko, N., Heller, N. H., Cheong, Y., & Smith, J. (2017). Persistent illusory apparent motion in sequences of uncorrelated random dots. *Journal of Vision*, *17*(3), 19. https://doi.org/10.1167/17.3.19

Ramachandran, V. S., & Anstis, S. M. (1985). Perceptual organization in multistable apparent motion. *Perception*, *14*(2), 135–143. https://doi.org/10.1068/p140135
